# Pelvic Control Characteristics During Static Balance in Patients with Multiple Sclerosis: A Novel Sensor-Based Study

**DOI:** 10.3390/jcm14113854

**Published:** 2025-05-30

**Authors:** Zofia Dzięcioł-Anikiej, Anna Kuryliszyn-Moskal, Alina Kułakowska, Janusz Dzięcioł, Mariusz Baumgart, Amanda Maria Kostro

**Affiliations:** 1 Department of Rehabilitation, Medical University of Białystok, 15-089 Białystok, Poland; anna.kuryliszyn-moskal@umb.edu.pl (A.K.-M.); amanda.kostro@umb.edu.pl (A.M.K.); 2 Laboratory of Physical Activity Clinical Research Center (CBK), Medical University of Białystok, 15-089 Białystok, Poland; 3 Neurology Clinic, Medical University of Białystok, 15-089 Białystok, Poland; alina.kulakowska@umb.edu.pl; 4 Department of Human Anatomy, Medical University of Białystok, 15-089 Białystok, Poland; janusz.dzieciol@umb.edu.pl; 5 Department of Normal Anatomy, Faculty of Medicine, Collegium Medicum in Bydgoszcz, Nicolaus Copernicus University in Toruń, 87-100 Toruń, Poland

**Keywords:** multiple sclerosis, posturography, balance, gait, accelerometer

## Abstract

**Background/Objectives:** Multiple sclerosis (MS) is an inflammatory and neurodegenerative disease of the central nervous system. MS lesions can affect the motor, sensory, and visual nerves, leading to impaired balance, muscle tension, and pain. The occurrence of the above can significantly affect quality of life. There is therefore a need to use objective methods of functional assessment for balance and gait disorders in patients with MS. The aim of the study was to assess the functional status and quality of life in people with multiple sclerosis with the simultaneous use of an accelerometer and baropodometric mat. **Methodology**: The research was conducted using functional tests: Tinetti test, Tandem Pivot Test, timed up and go test, and the Berg Balance Scale. In addition, the Sensor Medica baropodometric mat and the Baiobit balance and gait assessment system were used to objectively assess balance and gait. The assessment was performed once. The study involved 34 participants diagnosed with relapsing–remitting multiple sclerosis compared to a control group consisting of healthy individuals with similar demographic data to the study group. **Results**: Significant differences were found between the study and control groups in both functional and baropodometric assessments as well as when using an accelerometer in the pelvic area. **Conclusions**: Higher disturbances and differences are detected in the pelvic area; therefore, it is necessary to consider assessment using the simultaneous measurement of the displacement of the center of gravity located both on the pelvis and on the feet during the performance of different tasks—static and dynamic.

## 1. Introduction

Multiple sclerosis (MS) is a chronic, autoimmune disease of the central nervous system, susceptible to inflammatory, neurodegenerative, and demyelinating changes [1]. MS can affect the optic nerves, the brain, and the spinal cord. It is estimated that the number of people with MS worldwide ranges from 5 to 300 per 100,000 people and increases at higher latitudes. People affected by MS, compared to healthy populations, live much shorter—75.9 years in MS patients compared to 83.4 years without MS. The diagnosis is made based on a combination of symptoms, radiological findings, and laboratory test results (e.g., oligoclonal bands specific for the cerebrospinal fluid), which are components of the 2017 McDonald criteria [2,3]. The course of the disease varies and depends on the treatment undertaken. The most common form of MS, occurring in about 80% of patients, is relapsing–remitting multiple sclerosis (RRMS). It affects women three-times more frequently and is characterized by alternating periods of exacerbation and remission. After 10–15 years, RRMS may develop into secondary progressive multiple sclerosis (SPMS), which is characterized by the gradual development of the disease with the possibility of alternating periods of exacerbation and remission. In turn, in primary progressive multiple sclerosis (PPMS), the disease develops from the beginning of its appearance and there are no periods of relapse and remission. However, in progressive relapsing multiple sclerosis (PRMS), the disease progresses continuously with periods of relapse occurring. In PRMS, the patient’s condition may completely or partially return to the state before the relapse occurred [1,4,5]. Typical symptoms include sensory disturbances, motor weakness, gait impairment, lack of coordination and balance, optic neuritis, and the occurrence of Lhermitte’s sign (a sensation similar to an electric shock along the spine when the neck is bent). Other symptoms include urinary system dysfunction, intestinal dysfunction, and sexual dysfunction [6]. MS symptoms significantly affect functional ability and quality of life (QoL). Depression, anxiety, fatigue, and disability are among many factors that have been shown to reduce QoL in people with MS, especially in terms of social activity and work capacity [7,8,9,10].

Balance disorders increase with age and contribute significantly to increased disability. On the one hand, these disorders are associated with reduced physical activity and limitations in daily activities. On the other hand, the anxiety of falling also results in limited physical activity. Maintaining balance requires the cooperation of three systems: vestibular, visual, and proprioceptive. Aging and neurodegenerative diseases such as MS have consequences in the form of sensory–motor deficits, and in their course, there is damage to the nerves controlling muscles, leading to their dysfunction. A significant role in the treatment of balance disorders is played by physiotherapy, including targeted exercises and education on prevention and coping with falls [11]. The vast majority of MS patients have abnormalities in posture and gait control even in the early stages of the disease, and balance disorders affect 50–80% of MS patients, 50% of whom report at least one fall per year. Balance disorders in MS consist of several aspects: limited ability to maintain position, slowed movement, and delayed reactions to body displacement. In these patients, changes in walking are observed, which are characterized by a slower walking speed, balance disorders, and a reduced frequency of physical activity. Falls often cause injuries [12,13]. Decreased gait endurance, common in people with multiple sclerosis, leads, in addition to an increased risk of injury, to reduced social participation [14]. Studies also report that quantitative parameters defining balance deviations in MS are associated with BMI and age in both static and walking assessments, and therefore should be taken into account when assessing balance disorders in MS [12]. It has also been shown that patients with multiple sclerosis who experience balance problems, a slower walking speed, and greater fatigue are at high risk of falls. The relationship between walking speed and falls may be mediated by impaired balance and fatigue level. It seems reasonable to pay attention to the aspect of balance assessment and fatigue level when developing rehabilitation interventions, which may reduce the frequency of falls in people with MS [15]. Specialized devices measuring trunk sway are used in the diagnosis of gait and balance deficits in MS patients. Studies confirm that increased trunk oscillations are observed in MS, strongly correlated with their EDSS (Expanded Disability Status Scale) results, especially during tasks related to posture and gait [16,17,18]. This is also supplemented by tests assessing gait and balance function, such as the Berg Balance Scale (BBS), Dynamic Gait Index (DGI), and timed up and go (TUG). BBS, DGI, and TUG are reliable tests that can also be used in teleassessments, as well as when conducted face to face [19].

The literature indicates the high clinical value of gait assessment in patients with multiple sclerosis, which affects their daily life, causes a risk of falls, and limits activity. These limitations may relate to secondary psychological problems, such as depression or anxiety. By using appropriate personalized therapy, it is possible to improve balance and quality of life, which will contribute to the independence and increased functional activity of patients. Despite numerous studies on the mechanisms of these disorders, there are a limited number of studies that specifically examine the use of advanced technologies in their functional diagnostics and treatment. The current literature focuses mainly on traditional assessment methods, such as clinical tests or subjective questionnaires, which, although useful, may not fully capture the dynamic nature of balance and gait problems in MS patients [20]. Falls are a serious health problem for people with MS, and the early detection of impaired postural control is an important prognostic factor in fall prevention. The most precise method for determining these risks is laboratory technology, but it is expensive. The second example is clinical tests, but they are not precise and may not detect hidden disorders. An alternative solution is the use of accelerometry and baropodometric mats, because this method is inexpensive, ubiquitous, and portable [21]. It has been documented that people with MS experience impairments in gait and mobility, particularly related to the energetic costs of walking [22]. These variables cannot be fully captured by manually measured walking tests or rating scales administered at periodic clinical visits. Research emphasizes that attention should be paid to the assessment of walking ability, including the ability to turn while walking, in people with MS using accelerometers, e.g., smartphones [23]. As MS progresses, body posture is disturbed and the risk of falls increases [24]. Accelerometry sensors located on the sternum or lumbar spine are used in the diagnosis of gait and balance disorders in patients with MS [25]. These devices enable the real-world monitoring of patients and suggest biomarkers of symptoms and behaviors associated with underlying gait disorders [26].

Thus, there is a clear research gap: an integrated approach using modern, precise measurement tools that could provide more objective and detailed data on patients’ motor functioning are missing. The available literature confirms the lack of reports using the device in MS patients. However, the Baiobit system is used in the assessment of locomotor skills of athletes, or patients with cervical problems and a temporomandibular joint, and has been validated in locomotor patient assessment [27,28,29,30].

Modern technologies, such as Baiobit sensors and baropodometric mats, open up new possibilities in assessing patients’ motor functions. Baiobit sensors, thanks to advanced movement analysis algorithms, allow for precise recording of balance and gait dynamics parameters, such as stance phase time, step length, or gait rhythm variability. In turn, baropodometric mats provide detailed information on foot pressure distribution and postural stability during standing and movement.

The use of these tools in MS research is particularly promising, as it allows for a more accurate diagnosis of movement problems and an assessment of the effectiveness of therapeutic interventions. Additionally, the objectivity and repeatability of the results allow for monitoring of the patient’s progress over time, which is crucial in the rehabilitation process. Previous studies using similar technologies have focused mainly on other disease entities, such as Parkinson’s disease or stroke [24]. Introducing these tools to MS research is an innovative approach that can contribute to a better understanding of the characteristics of gait and balance disorders in this group of patients. Therefore, there is a need for early objective and reliable diagnosis of balance and gait disorders in MS patients.

## 2. Aim of Study

The aim of this study is to assess the usefulness of functional tests and innovative tools such as Baiobit sensors and baropodometric mats in diagnosing and monitoring balance and gait parameters such as foot load, step length, step speed, swing length, surface area of the center of the gravity ellipse, or the speed of its movement in patients with multiple sclerosis compared to healthy people. We also aim to explore how objective data obtained using these technologies can be used in clinical practice involving patients with MS.

## 3. Methodology

This study was conducted after obtaining the consent of the Bioethics Committee of the Medical University of Białystok No. R-I-002/392/2019 at the Rehabilitation Clinic of the Medical University of Białystok.

The study included patients diagnosed with relapsing–remitting multiple sclerosis according to the 2017 McDonald criteria, who had not had an MS attack in the last 3 months and whose EDSS score was <5. Patients with a confirmed diagnosis at least one year ago qualified for the study. According to the inclusion criteria, patients should not have proprioceptive ataxia or cognitive problems with concentration and memory.

The study excluded people who had diseases other than MS that impaired gait and balance, as well as people who had undergone gynecological or orthopedic surgery of the lower limbs and/or spine, and people who had suffered injuries to the joints of the lower limbs and/or spine. Participants were presented with the methodology and possible side effects (injuries related to exertion or MS attack), after which each of them signed an informed consent form to participate in the study. Finally, through qualification, 34 patients were included—24 women and 10 men. Demographic data of the group are presented in Table 1. The age of the patients ranged from 47 to 59 years, with an average age of 53 years. All patients were right-handed. The level of physical activity in both groups was the same; all participants were not active in leisure activities. The patients’ body mass index (BMI) values were in the range of 20.07–29.63, which indicated that the patients were not obese (Table 1). The inclusion criteria for the control group included, among others, independent walking, no comorbidities affecting walking ability, no occurrence of balance disorders, and no injuries or surgical procedures in the lower limbs that could impair walking.

Then, all participants from both groups were functionally assessed using tests such as the Berg Scale, Tinetti test, Tandem Pivot Test, and up and go test with right rotation.

### 3.1. Berg Balance Scale (BBS)

The test allows for the assessment of static and dynamic balance. It contains 14 movement tasks, and each task is assessed on a five-point scale (0–4). A total of 54 points can be obtained. A score from 0 to 20 points indicates complete dependence on a wheelchair. From 21 to 40 points are awarded to a patient who moves with assistance. An independent patient should score from 41 to 56 points. The tasks include changing position from sitting to standing, standing without assistance, sitting without support, changing position from standing to sitting, moving from a sitting position, standing with eyes closed, standing at attention, picking up objects from the floor, standing on one leg, torso twists with fixed feet, reaching forward in a standing position, turning 360 degrees, stepping on a step, and standing with feet in a line (Tandem Stance Test). During this test, the patient can use orthopedic aids.

### 3.2. Tinetti Test

This is a scale assessing static and dynamic balance. It consists of two parts: the first assesses gait, the second assesses balance. The patient can receive a maximum of 12 points from the gait assessment section, while the balance assessment section can receive 16 points. The maximum possible number of points to be obtained from both parts of the test is 28. Studies have shown that obtaining a score below 26 points is associated with an increased risk of falls. The test takes about 10 min to complete.

### 3.3. Tandem Pivot Test—180° Rotation Test

This test is used to assess coordination and static and dynamic balance, which in turn is associated with assessing the risk of falls. During this test, the patient must assume a tandem position, which means that the feet should be positioned one behind the other, with the toes of the forward foot touching the heel of the forward foot. The patient then rotates 180°. A six-point scale is adopted (from 0 to 5), with 0 meaning that the patient is unable to perform the task, and 5 indicating that the task was performed correctly, completely independently.

### 3.4. Timed Up and Go Test (TUG)

TUG is another frequently used test to assess the risk of falls. According to this test, on command, the patient stands up from a chair (seat height should be about 46 cm), walks at a safe pace to a line 3 m away, turns around and returns to the chair and sits down on it. Time is measured from the moment of standing up from the chair to the moment of taking a sitting position in the chair again. The use of orthopedic aids (a cane or a walker) is allowed. The shorter the time, the better the patient’s functional efficiency. A time of less than 13.5 s indicates an increased risk of falls. Additionally, the number of steps taken during the test was counted, including the number of steps during the turn.

### 3.5. Baropodometric and Motion Assessment

Baropodometric assessment was also performed on the Sensor Medica Free Step mat (Guidonia Montecelio, Italy), including static, dynamic, and Romberg tests with eyes closed and open for 60 s. At the same time, during the Romberg test, the participants had the Baiobit motion sensor attached to the sacrum and lumbar spine, which allowed for simultaneous measurement of the displacement of the center of gravity in the feet and pelvis during posturographic tests. This is an innovative approach to assessing balance disorders using the synchronization of both devices. In addition to the posturographic assessment, the Baiobit sensor was also used to assess gait over a distance of 6 m and to perform the up and go test.

### 3.6. Static Baropodometry

This test consisted of standing for 30 s with the limbs along the torso and the feet placed hip-width apart.

### 3.7. Dynamic Baropodometry

The test consisted of walking several times on a designated mat in 1 min.

### 3.8. The Posturography—Romberg’s Test

Romberg test consisted of standing for 60 s with the feet hip-width apart and the upper limbs bent to 90 degrees in the shoulder joint with the palm facing the floor. The test was performed twice—with eyes closed and open.

The data obtained were processed using Microsoft Excel 365. Statistical analysis was performed using the Mann–Whitney test in Statistica 13, and the significance level was *p* = 0.05. Due to the sample size of less than 100 observations, non-parametric tests were used for the analysis. This concern was also confirmed by the assessment of the normality of the distribution using the Shapiro-Wilk test.

## 4. Results

In the assessment of quality of life concerning symptoms in the study group, the majority of respondents complained about the presence of a feeling of heaviness in the legs. Other common symptoms were as follows: fatigue and balance disorders. Pain was also a fairly common problem. Additionally, respondents observed numbness, depression, superficial sensation disorders, sphincter disorders, sleep disorders, stiffness, spasms, paresis and nystagmus, strength disorders, and intention tremor. Balance disorders were reported by 13% and 40% as very frequent and frequent.

In the assessment of differences between groups, statistical significance was obtained in all functional tests assessed. The parameters were assessed using statistical tests and the values were obtained as described in the tables below, where M—mean, Me—median, SD—standard deviation, Min—minimum, Max—maximum, *p*—*p*-value. *p*-value after Bonferroni correction is additionally marked with the symbol (*). Due to the fact that non-parametric tests were used, the medians in both groups were compared and their clinical significance was determined in the Results Section. MS patients achieved worse results compared to the control group. The differences are presented in Table 2.

Patients needed almost twice as long to perform the TUG test and completed a greater number of steps compared to the study group. The Me in the control group was 5.98 and in the study group it was 10.05 (*p* < 0.001). Patients with MS took more steps, 17, compared to the control group at 11 (*p* < 0.001), and the turnover time in patients with MS was 6 s, compared to 3 in the control group (*p* < 0.001). Moreover, MS patients were unable to perform the Tandem Pivot Test—180° rotation test. Most often, the result was 0, which indicates major problems with balance in the clinical assessment. Comparing the control group, the test results were most often 5 (*p* < 0.001). In turn, according to Tinetti, MS patients received scores below 26 points (the median was 20, *p* < 0.001), which also indicates the risk of falling as a result of balance disorders. When analyzing the results, MS patients need help in moving compared to the healthy group in the BBS assessment—median of 37 points *p* < 0.001. In the functional assessment, the group of patients diagnosed with MS obtained worse results in the assessment of barometric static measurements compared to the control group in the assessment of foot load and foot surface. A greater load and surface area of the forefoot and lower load on the hindfoot, as well as average and maximum load on the lower limbs, were observed in patients with multiple sclerosis compared to healthy people. This suggests shifting the center of gravity further forward, which increases the surface area of the forefoot and, consequently, reduces the load on the heels and the overall load on the lower limbs. At the assumed level of statistical similarity (*p* < 0.05), a higher left foot surface (58 cm^2^) was observed in the study group than in the control group (46 cm^2^). Moreover, higher results were obtained for the forefoot of both feet in the study group (right—29 cm^2^, left—27 cm^2^) than in the group of healthy people (21 cm^2^—right, 18 cm^2^—left). Patients with MS loaded their lower extremities to a lesser extent (left hindfoot loading—68%, right hindfoot loading—68%) compared to the study group (left hindfoot loading—83%, right hindfoot loading—81%). The results of the maximum load of both feet and the average load of both feet were also lower. The results are presented in Table 3. Dynamic baropodometry, although the surface areas of the feet in people with MS differ from those in the control group, showed only that the surface area of the left foot in the study group is higher (101 cm^2^) than in the control group (79 cm^2^). This suggests a lowering of the left foot arch or incorrect control of the “worse” side according to the study participants; all declared right-sided dominance. The load on the left foot was also statistically significantly lower in patients with MS (3056 gr/cm^2^) (Table 4).

In the posturographic assessment on the baropodometric mat, significant statistical differences were obtained between parameters such as the area of the ellipse, the position of the center of gravity on the X- and Y-axes for both closed and open eyes, and in the case of the latter, significant differences were obtained in the speed of the center of gravity movement. People with MS showed a larger area of the ellipse of the center of gravity in the foot area (192.36 cm with open eyes and 2489.21 cm with closed eyes) and a higher speed of its movement (46.7 mm/s with open eyes and 46.15 mm/s with closed eyes). The values of X and Y were also higher than in the control group. This fact indicates a worse ability to maintain balance in the assessment of the foot position (Table 5 and Table 6).

The most statistically significant differences were obtained when assessing the gait of people with MS with the Baiobit accelerometer compared to healthy people. The results show that patients have worse gait symmetry (Me = 76.4), move slower (Me = 0.83), take shorter steps (Me = 1.28:1.45 Me = 1.21:1.47), and take fewer steps (Me= 4:5, Me = 4:6), but above all, their pelvis position is different than in healthy people. People with MS move their pelvis more anteriorly. This is also confirmed by the results of the baropodometric mat study. In addition, they rotate it maximally to the left and minimally to the right. The pelvis of people with MS lowers more to the left and minimally to the right. In the control group, this position is the opposite. Detailed information is provided in Table 7.

Similar to the assessment of gait using Baiobit, a significant number of variables indicating balance disorders in people with MS were obtained in the posturographic assessment. Patients with both closed and open eyes show a larger elliptical surface, higher anteroposterior and medial–lateral oscillations at a much higher speed of their movement, and almost twice as long a center of gravity path compared to healthy people. This suggests that the balance examination of MS patients should also assess the center of gravity path in the pelvic area. Detailed information is provided in Table 8 and Table 9.

In turn, in the TUG assessment using Baiobit, people with MS showed worse times during rising, turning, returning, final turning, and sitting. Anteroposterior and lateral acceleration were also lower during rising. This indicates a worse condition of patients who need more time to complete the task. Detailed information is provided in Table 10.

## 5. Discussion

Balance disorders are a common symptom in people with multiple sclerosis. They are considered to be among the symptoms that significantly limit an individual’s independence. In addition to poor balance, fatigue, spasticity, and muscle weakness also contribute to the increased risk of falls. A predisposition to falls may occur even before balance disorders become noticeable [31]. Studies confirm that despite many variables and a lack of standardized reporting, data synthesis is difficult. However, researchers believe that people with MS show significant deficits in postural control compared to healthy individuals, regardless of the type of task and its complexity [32].

In turn, many studies report the use of measuring devices in telemedicine to monitor and counteract health threats, including multiple sclerosis. Wearable devices enable real-world monitoring of patients and suggest biomarkers of symptoms and behaviors related to basic gait disorders. Accelerometers and baropodographic mats are most often used for this purpose [12,21,23,26].

Additionally, in the work of Özden, at all analyses of the performance of the use of people differed by the timed up and go (TUG) test and the Five-Time-Sit-to-Stand (FTSTS) test. The reaction time to step (SRT) of people was calculated by the built-in software on the video. The time between the instructions to take a step and the first contact of the feet with the ground in the first step was recorded. It was proven that people with MS have a reduced SRT. It does not cause failures in performance. In addition, the Activities-specific Balance Confidence (ABC) scale, the 12-item Multiple Sclerosis Walking Scale (MSWS-12v2), and the Falls Efficacy Scale International (FES-I) were used. Significant differences were found between groups in SRT, FES-I, ABC, and FTSTS (*p* < 0.05). There was no significant correlation between SRT and any other parameter (*p* > 0.05). TUG was moderately correlated with MSWS-12 and FES-I (r 1 = 0.426, r 2 = 0.495, *p* < 0.05). In addition, there was a moderate correlation between ABC and TUG and FTSTS (r 1 = −0.605, r 2 = −0.468, *p* < 0.05) [33].

In our work, we obtained the difference in steps when assessed using the Baiobit accelerometer. We used the Baiobit to assess TUG, as well as the traditional TUG test. We obtained, similar to the above study, differences between the groups in the assessment of time, steps, and the time needed for rotation (*p* < 0.05), while in the TUG test using the Baiobit accelerometer we obtained differences between groups for the transition time and at the decisive moments of return, final rotation, and when sitting on the chair (*p* < 0.05).

For example, Brincks et al. evaluated parameters of balance using stabilometric tests, TUG and T25FW; however, there is no correlation between balance and walking performance. In this study, the walking performance of 13 people with MS (Expanded Disability Status Scale = 2.5) and 13 healthy controls was assessed using fast walking pace, timed up and go, and timed 25-foot walk. Postural balance was measured using stabilometry, a 95% confidence interval of sway area ellipse, and sway velocity. Apart from sway velocity (*p* = 0.07), significant differences were found between people with MS and healthy controls in postural balance and walking [34].

We observed differences in static parameters on the baropodometric matrix and in the case of using an accelerometer. We also added functional elements of TUG and use of the Baiobit device—as a result, we obtained additional differences. We obtained the leading differences in the examination on the baropodographic mat in the posturographic assessment with the Romberg test with closed and open eyes for parameters such as the area of the ellipse, or the position of the center of gravity in relation to the X- and Y-axes. At the same time, we assessed balance disorders in the pelvis using Baiobit and here we obtained more differences between the assessed groups, including in the assessment of the area of the ellipse in the 95% range, anterior–posterior and medial–lateral sway, or the speed of the center of gravity movement. This confirms the fact that balance and stabilization disorders are visible in the pelvic area in people with MS. A posturographic examination should be performed using both an accelerometer to assess the pelvis and a baropodometric mat to assess the pressure of the feet on the ground.

In the studies of Dujmovic et al. [35], it was shown that the gait of patients with MS differs from the typical gait pattern of society. Spatiotemporal gait pattern characteristics were analyzed in patients with relapsing–remitting (RR, n = 52) and primary progressive (PP, n = 18) MS compared to age-matched healthy controls (HC, n = 40). The study was performed on an electronic GAITRite track with an active area of 5.5 m. The cycle time (CT), step length (SL), swing time (ST), double support time (DST), gait speed (GV), and calculated symmetry index (SI) for CT, SL, and ST were measured. Although PPMS patients were significantly different from RRMS patients in most gait parameters, a subgroup analysis of patients matched for age and disability (Expanded Disability Status Scale Score -EDSS, 3.0–5.0) showed a similar gait performance in RRMS and PPMS patients with the same level of disability, except for CT and ST symmetry parameters, which were more impaired in the PPMS group. The EDSS score correlated significantly with CT, DST, SL, and GV, but no significant correlation was found with ST.

The results are consistent with our studies. We obtained statistically significant differences in the assessment of symmetry and gait speed using the Baiobit device (*p* < 0.05).

In studies, it was shown that gait analysis launched on the biological mechanism supports the clinical assessment, which regards the gait seen even in the first occurring stages of MS, first during fast walking. Step length and gait speed were identified as the most important clinical gait measurements. Therefore, gait analysis can be based on supporting the treatment of MS patients with health impairment in the future, e.g., for a more detailed classification of the unassigned [36].

The gait and balance assessment that we also performed, including two systems and a functional assessment, confirms the fact that balance and gait disorders are observed in the MS group. In the study, we found that the combination of baropodometric and accelerometric methods is the principle for gait and balance disorders in MS members. However, further studies using precise biomechanical systems are needed on this matter.

To apply accelerometry solutions in an outpatient setting to characteristic spatiotemporal gait parameters in people with MS, Pau performed the eliminated 25-foot walk test (T25FW) on 105 people with MS using a commercially available accelerometer. All objectively determined parameters, except step, were significantly impaired in the MS group. This confirms the importance of early studies of locomotor disorders for individual therapy of MS patients [37].

Similar observations were also obtained in the utility version using Baiobit accelerometers.

The obtained results confirm the research hypothesis. MS patients have balance and gait disorders compared to the control group, and these disorders are measured both at the level of the pelvis and feet. Therefore, the simultaneous use of an accelerometer and a baropodography mat enables precise diagnosis of balance and gait disorders in patients with MS.

The results of this study confirm that patients with multiple sclerosis have significant impairments in pelvic control that translate into reduced postural stability. Unlike previous studies that generally described balance problems in this group of patients, our study focuses on the role of the pelvis as a key element of postural control and its specific dysfunctions.

In a comparative analysis with the previous literature, it can be seen that previous studies have mainly focused on the control of the trunk or lower limbs, neglecting the importance of the pelvis as a dynamic link between the upper and lower body. Our results indicate that MS patients have difficulty maintaining a neutral pelvic position during tasks requiring balance control, which may affect their ability to compensate for balance disorders and increase the risk of falls.

The novelty of the study is the use of tools that allow for precise assessment of pelvic control in dynamic conditions. We indicate that not only is general postural control impaired, but that these disorders originate from functional limitations of the pelvic region. This may be of practical importance when planning therapy—suggesting the need to include targeted interventions to strengthen pelvic control, and not just general balance exercises.

### Limitation, Recommendations, and Generalizations

The conducted studies had both limitations and advantages. The limitations include the fact that only results from a small group of patients were presented. This is the first preliminary study using the Baiobit motion sensor while using the Sensor Medica baropodometric mat in gait and balance assessment, which is a preliminary report for further studies with a larger number of participants and more variables. We will certainly use the assessment in MS patients with a different functional status in the future, so the sample and number of variables will be closer to the intended calculations. In this study, we wanted to have as uniform a study group as possible to evaluate the use of systems for assessing balance disorders in people with MS. The presented results can be used in the practice of both interdisciplinary medical teams to detect balance disorders in people with MS.

## 6. Conclusions

Based on the collected materials and our own results, it can be stated that MS patients have balance disorders measured in both functional and posturographic tests using an accelerometer and a baropodometric mat. Higher disorder and differences are detected in the pelvic area; therefore, an assessment using simultaneous measurement of the displacement of the center of gravity located both on the pelvis and on the feet during the performance of various tasks—static and dynamic—should be considered. The research should be expanded to include other groups of patients.

## Figures and Tables

**Table 1:** Demographic data of study group.

	MS Group						Control Group						
	n	Me	Min	Max	Q1	Q3	n	Me	Min	Max	Q1	Q3	*p*
**Age**	34	55	47	59	50	56	34	56	45	59	50	57	0.822
**BMI**	34	24.8	20.1	29.6	22.5	27.2	34	24.8	22.1	28.4	22.5	27.2	0.849
**EDSS**	34	3	1	4	2	3	0	-	-	-	-	-	-
**Duration of illness**	34	4	2	5	3	4	0	-	-	-	-	-	-

BMI—body mass index, EDSS—Expanded Disability Status Scale, n—numbers, Q1—quartile 1, Q3—quartile 3, Me—median, Min—minimum, Max—maximum, *p*—*p*-value.

**Table 2:** Comparison of the control and study groups in functional tests.

	Control Group					Study Group					*p*
	Q1	Q3	Min	Me	Max	Q1	Q3	Min	Me	Max	
**TUG Time**	5.3	6.2	4.6	5.9	6.9	9	15.7	7.2	10	21.9	* 0.001
**TUG Steps**	10	12	9	11	12	15	20	12	17	29	* 0.001
**TUG Rotation**	3	4	3	3	4	4	7	3	6	8	* 0.001
**BBS**	56	56	56	56	56	33	43	17	37	47	* 0.001
**Tandem Pivot 180**	4	5	4	5	5	0	2	0	0	4	* 0.001
**Tinetti Balance**	16	16	16	16	16	10	15	4	12	15	* 0.001
**Tinetti Gait**	11	12	11	12	12	6	9	5	8	10	* 0.001
**Tinetti Total**	27	28	27	28	28	16	23	9	20	25	* 0.001

TUG—timed up and go test, BBS—Berg Balance Scale, Q1—quartile 1, Q3—quartile 3, Me—median, Min—minimum, Max—maximum, *p*—*p*-value, * Bonferroni correction.

**Table 3:** Comparison of static baropodometry assessment.

	Control Group					Study Group					*p*
	Q1	Me	Min	Max	Q3	Q1	Me	Min	Max	Q3	
**Left foot surface cm^2^**	44	46	35	53	48	51	58	30	92	68	0.006
**Right foot surface cm^2^**	39	48	30	62	56	46	57	35	80	70	0.056
**Left forefoot surface cm^2^**	15	18	12	28	20	20	27	12	57	39	0.005
**Right forefoot surface cm^2^**	16	21	7	38	30	23	29	9	54	34	0.034
**Left hindfoot surface cm^2^**	25	26	23	34	29	26	31	9	41	35	0.079
**Right hindfoot surface cm^2^**	23	26	22	33	30	22	26	17	49	30	0.863
**Left foot loading %**	49	52	42	62	54	44	50	36	66	55	0.863
**Right foot loading %**	46	48	38	58	51	45	50	34	64	56	0.863
**Left forefoot loading %**	12	17	9	36	22	23	32	6	70	47	0.003
**Right forefoot loading %**	14	19	6	52	38	26	32	8	91	40	0.048
**Left hindfoot loading %**	78	83	64	91	88	53	68	30	94	77	0.003
**Right hindfoot loading %**	62	81	48	94	86	60	68	9	92	74	0.048
**Max. loading of left foot gr/cm^2^**	1487	1612	1419	2261	2041	1383	1417	831	1775	1532	* 0.001
**Max. loading of right foot gr/cm^2^**	1463	1631	1368	2049	1868	1257	1445	912	1729	1645	0.013
**Mean loading of left foot gr/cm^2^**	638	687	630	896	716	479	553	341	1027	664	0.003
**Mean loading of right foot gr/cm^2^**	557	642	556	834	742	492	551	450	806	658	0.027

Q1—quartile 1, Q3—quartile 3, Me—median, Min—minimum, Max—maximum, *p*—*p*-value, * Bonferroni correction.

**Table 4:** Comparison of dynamic baropodometry assessment.

	Control Group					Study Group					*p*
	Q1	Me	Min	Max	Q3	Q1	Me	Min	Max	Q3	
**Left foot surface cm^2^**	230	79	65	90	250	220	101	0	138	260	0.010
**Right foot surface cm^2^**	230	86	66	95	240	230	92	69	144	250	0.091
**Max. loading of left foot gr/cm^2^**	197	3272	2988	3480	216	200	3056	0	4236	225	0.027
**Max. loading of right foot gr/cm^2^**	205	3076	2296	3636	215	189	2892	2252	4604	227	0.679
**Mean loading of left foot gr/cm^2^**	77	1076	947	1168	83	82	949	0	1194	111	0.054
**Mean loading of right foot gr/cm^2^**	79	997	833	1169	91	84	947	758	1399	104	0.408
**Left forefoot loading %**	3180	58	46	63	3332	2776	59	25	70	3156	0.449
**Right forefoot loading %**	2752	60	47	67	3316	2832	61	38	80	3472	0.630
**Left hindfoot loading %**	1011	42	37	54	1133	765	41	30	75	1059	0.449
**Right hindfoot loading %**	935	40	33	53	1100	903	39	20	62	1066	0.630
**Left foot loading in the medial part %**	53	53	35	68	61	50	54	34	60	65	0.449
**Left foot loading in the lateral part %**	55	47	32	65	64	58	46	40	66	67	0.449
**Right foot loading in the medial part %**	39	53	40	60	47	35	51	30	65	50	0.809
**Right foot loading in the lateral part %**	36	47	40	60	45	33	49	35	70	42	0.809

Q1—quartile 1, Q3—quartile 3, Me—median, Min—minimum, Max—maximum, *p*—*p*-value.

**Table 5:** Comparison of open-eye Romberg baropodometry assessment.

	Control Group					Study Group					*p*
	Q1	Me	Min	Max	Q3	Q1	Me	Min	Max	Q3	
**Ellipse area cm**	32.1	51.6	14.3	99.2	62.4	136.9	192.4	32.1	3486.4	789.6	* 0.001
**Swing length mm**	1131.6	1310.8	1014.4	1929.0	1793.9	1236.6	1406.3	1016.3	4628.7	1960.9	0.270
**X-axis**	6.5	8.8	4.4	11.6	9.6	13.3	17.6	6.5	67.3	34.5	* 0.001
**Y-axis**	6.3	7.9	4.1	10.9	8.1	12.7	14.4	6.3	65.9	28.5	* 0.001
**Delta X**	7.3	9.2	5.4	11.7	10.5	11.8	15.9	7.3	58.8	27.1	* 0.001
**Delta Y**	7.6	8.6	4.2	17.4	11.2	11.6	17.6	8.6	72.6	28.9	* 0.001
**Maximum sways**	1.3	1.5	1.3	1.8	1.7	1.6	1.8	1.3	3.5	2.6	* 0.002
**Minimal sways**	0	0	0	0.02	0.01	0	0.01	0	0.01	0.01	0.630
**Average speed mm/s**	33.6	35.9	29.4	43.4	37.5	40.4	46.7	33.1	91.0	56.5	* 0.001
**Mean position in X-axis**	−0.2	3.3	−23.2	13.8	9.6	−8.1	−3.1	−38.1	20.9	9.1	0.582
**Mean position in Y-axis**	−28.3	−24.8	−39.8	−9.9	−12.1	−48.6	−37.1	−67.8	12.0	−24.8	* 0.016

Q1—quartile 1, Q3—quartile 3, Me—median, Min—minimum, Max—maximum, *p*—*p*-value, * Bonferroni correction.

**Table 6:** Comparison of closed-eye Romberg baropodometry assessment.

	Control Group					Study Group					*p*
	Q1	Me	Min	Max	Q3	Q1	Me	Min	Max	Q3	
**Ellipse area cm**	111.3	210.1	62.4	466.7	261.4	928.8	2489.2	133.2	4064.5	2910.2	* 0.001
**Swing length mm**	1470.3	1840.4	1106.4	2725.9	2283.3	1306.9	1453.9	1040.5	3077.4	1743.9	0.179
**X-axis**	12.9	16.6	9.6	25.6	19.7	34.4	59.1	13.0	106.7	67.3	* 0.001
**Y-axis**	10.9	14.7	8.3	23.2	18.2	27.6	48.5	13.0	65.9	53.7	* 0.001
**Delta X**	13.7	17.4	9.2	27.6	22.6	28.6	46.6	13.4	109.7	53.3	* 0.001
**Delta Y**	16.4	17.3	11.1	27.6	19.7	31.5	51.2	14.3	83.0	62.9	* 0.001
**Maximum sways**	1.8	2.1	1.4	8.7	2.7	1.9	2.6	1.5	6.1	3.8	0.228
**Minimal sways**	0	0	0	0.020	0	0	0.010	0	0.020	0.010	0.102
**Average speed mm/s**	37.8	44.3	31.6	61.1	49.5	41.2	46.1	34.2	85.8	55.8	0.286
**Mean position in X-axis**	3.3	11.2	−16.3	37.1	15.8	0.1	10.2	−17.7	53.8	16.9	0.535
**Mean position in Y-axis**	−32.6	−21.5	−44.6	−12.3	−19.5	−49.5	−37.4	−80.1	12.9	−22.3	0.063

Q1—quartile 1, Q3—quartile 3, Me—median, Min—minimum, Max—maximum, *p*—*p*-value, * Bonferroni correction.

**Table 7:** Comparison of gait in Baiobit accelerometer assessment.

	Control Group					Study Group					*p*
	Q1	Me	Min	Max	Q3	Q1	Me	Min	Max	Q3	
**Symmetry**	94.4	96	90.6	99.3	98.3	65.9	76.4	16.6	90.2	80.8	* 0.001
**Walking speed**	1.1	1.2	0.8	1.6	1.4	0.7	0.8	0.6	1.6	1.1	* 0.001
**Rhythm**	109.8	115.2	88.8	132.7	126.6	101.7	106	89.8	131.9	118.2	0.153
**Left foot propulsion**	6.9	9.1	3.6	14.8	10.1	4.4	5.7	2.2	47.9	9.1	0.125
**Right foot propulsion**	6.3	8.9	4.1	14.4	10.2	3.9	7.3	2.2	24.1	10.3	0.256
**Left step length**	0.3	1.4	0.1	2.0	0.5	0.3	1.3	0.8	1.7	0.9	0.036
**Right step length**	5.4	1.4	0.9	1.9	7.3	4.7	1.2	0.8	1.9	6.530	0.017
**L step length % of height**	1.2	81	61	116	1.6	1.0	68	46	102	1.4	0.034
***p* step length % of height**	1.3	80	61	111	1.7	1.0	71	46	114	1.4	0.014
**Left step length % of height**	77	0.7	0.5	1	96	61	0.6	0.4	0.9	90	0.026
**Right step length % of height**	77	0.7	0.4	0.9	97	63	0.6	0.4	0.9	82	0.015
**Left step time**	0.6	1.1	0.9	1.9	0.9	0.6	1.1	0.9	1.5	0.7	0.242
**Right step time**	0.6	1.1	0.9	1.9	0.8	0.5	1.1	1.0	1.5	0.7	0.502
**Number of steps of the left foot**	1.0	5	4	25	1.2	1.1	4	2	13	1.270	0.036
**Number of steps of the right foot**	1.1	6	4	27	1.2	1.1	4	2	13	1.3	0.014
**Minimal pelvic tilt**	88	6	−8	19	98	70	10	−7	35	94	0.015
**Maximal pelvic tilt**	88	5	−8	24	97	74	6	−13	25	96	0.904
**Minimal pelvic obliquity**	5	−3	−8	3	17	3	3	−8	7	6	0.003
**Maximal pelvic obliquity**	5	3	−3	9	15	3	−3	−7	9	6	0.004
**Minimal pelvic rotation**	60.5	−5	−11	8	65.8	59.2	6	−8	27	74.8	0.003
**Maximal pelvic rotation**	60.7	5	−7	11	66.2	58.9	−6	−29	8	67.9	0.004

Q1—quartile 1, Q3—quartile 3, Me—median, Min—minimum, Max—maximum, *p*—*p*-value, * Bonferroni correction.

**Table 8:** Comparison of open-eye Romberg in Baiobit accelerometer assessment.

	Control Group					Study Group					*p*
	Q1	Me	Min	Max	Q3	Q1	Me	Min	Max	Q3	
**Area of the 95% confidence ellipse**	29	37	18	116	59	153	406	56	1104	594	* 0.001
**Anterior–posterior oscillation (mm)**	12	15	10	25	21	25	37	10	74	46	* 0.001
**Medial–lateral oscillation (mm)**	2	3	2	8	7	7	9	5	30	15	* 0.001
**Anterior–posterior oscillation speed (mm/s)**	2.9	3.8	1.9	5.3	4.3	5.9	7.5	3.2	18.9	10.4	* 0.001
**Medial–lateral oscillation speed (mm/s)**	0.9	1.1	0.5	2.6	1.2	1.9	2.7	1.4	5.7	3.8	* 0.001
**Path total length (mm)**	130	189	63	342	253	232	323	113	605	451	* 0.001
**LFS Balance Index**	3.1	4.2	2.2	7.8	5.5	0.7	1.1	0.3	2.1	1.7	* 0.001
**Mean pelvic tilt**	6	11	0	26	16	4	10	0	21	14	0.491
**Mean pelvic obliquity**	0	0	0	0	0	0	0	0	0	0	0.986
**Mean pelvic rotation**	0	0	0	1	0	0	0	0	2	1	0.139

Q1—quartile 1, Q3—quartile 3, Me—median, Min—minimum, Max—maximum, *p*—*p*-value, * Bonferroni correction.

**Table 9:** Comparison of closed-eye Romberg in Baiobit accelerometer assessment.

	Control Group					Study Group					*p*
	Q1	Me	Min	Max	Q3	Q1	Me	Min	Max	Q3	
**Area of the 95% confidence ellipse**	37	71	9	173	115	244	554	65	1991	964	* 0.001
**Anterior–posterior oscillation (mm)**	15	19	9	36	24	30	43	11	164	51	* 0.001
**Medial–lateral oscillation (mm)**	3	5	1	10	6	7	17	5	59	25	* 0.001
**Anterior–posterior oscillation speed (mm/s)**	3.7	4.5	2.4	6.2	5.0	6.9	10.3	2.7	27.6	12.3	* 0.001
**Medial–lateral oscillation speed (mm/s)**	1.1	1.4	0.6	2.2	1.6	2.2	3.2	1.5	14.1	5	* 0.001
**Path total length (mm)**	157	190	76	404	289	318	371	98	877	535	* 0.001
**LFS Balance Index**	2.2	2.9	0.9	7.6	5.1	0.6	0.9	0.3	2.5	1.5	* 0.001
**Mean pelvic tilt**	5	11	0	28	16	5	11	0	17	15	0.524
**Mean pelvic obliquity**	0	0	0	0	0	0	0	0	1	0	0.783
**Mean pelvic rotation**	0	0	0	1	0	0	1	0	7	1	0.011

Q1—quartile 1, Q3—quartile 3, Me—median, Min—minimum, Max—maximum, *p*—*p*-value, * Bonferroni correction.

**Table 10:** Comparison of TUG in Baiobit accelerometer assessment.

	Control Group					Study Group					*p*
	Q1	Me	Min	Max	Q3	Q1	Me	Min	Max	Q3	
**Time**	5.7	6.9	4.9	8.8	7.9	10.6	10.9	8.9	14.9	11.9	* 0.001
**Getting up time**	0.8	1.3	0.7	4.1	1.6	1.1	1.4	0.7	2.4	1.7	0.705
**Walking time**	0.5	1.2	0.4	1.7	1.3	1.9	2.8	0.6	4.0	3.9	* 0.001
**Half rotation time**	1.2	1.3	1.1	1.7	1.5	1.6	1.9	1.6	2.3	2.3	* 0.001
**Return time**	1.2	1.4	1.1	1.8	1.7	2.4	2.8	1.9	3.5	3.4	* 0.001
**Final rotation time**	0.7	0.9	0.6	1.4	1.1	1.2	1.4	1.1	2.5	1.5	* 0.001
**Sitting time**	0.5	0.7	0.2	0.9	0.8	0.6	0.9	0.4	2.3	1.1	0.007
**Lifting vertical acceleration**	0.4	2	0.1	7.6	3.3	0.8	1.5	0.5	2.3	2.1	0.863
**Lifting acceleration front–back**	5.5	6.2	0	15.6	8.2	1.7	2.6	1.0	6.4	4.5	0.012
**Lifting lateral acceleration**	0.7	1.2	0	3.5	2.5	0.6	0.6	0.3	0.9	0.9	0.017
**Sitting vertical acceleration**	2.3	8.3	0	18.4	9.2	3.8	4.5	1.0	7.9	6.8	0.121
**Sitting down, acceleration front-back**	4.4	5.4	0	10.8	6.2	1.2	2.9	0.3	8.5	4.5	0.105
**Sitting lateral acceleration**	0.5	0.8	0	3.9	1.4	0.8	1.1	0.5	2.3	1.7	0.242

Q1—quartile 1, Q3—quartile 3, Me—median, Min—minimum, Max—maximum, *p*—*p*-value, * Bonferroni correction.

## Data Availability

The data presented in this study will be made available at the request of the corresponding author due to the limitations of RODO data availability.
